# Positioning Digital Tracing Applications in the Management of the COVID-19 Pandemic in France

**DOI:** 10.2196/27301

**Published:** 2021-10-07

**Authors:** Marion Albouy-Llaty, Caroline Martin, Daniel Benamouzig, Eric Bothorel, Gilles Munier, Catherine Simonin, Jean-Louis Guéant, Emmanuel Rusch

**Affiliations:** 1 French Public Health Society Paris France; 2 Clinical Investigation Centre of Poitiers University Hospital 1402, Inserm Poitiers France; 3 Faculty of Medicine and Pharmacy University of Poitiers Poitiers France; 4 National Consultative Ethics Committee for Health and Life Sciences Paris France; 5 Conseil d’Etat Paris France; 6 French Covid-19 Scientific Council Paris France; 7 Sciences Po, Center for the Sociology of Organizations Paris France; 8 Assemblée Nationale (French Parliament) Paris France; 9 National Council of the Physicians’s College Paris France; 10 France-Assos Santé Paris France; 11 National Council of Medical Biology Paris France; 12 University of Lorraine, Inserm Unit 1256 Nancy France; 13 National Health Conference Paris France; 14 Unit Education, Ethics and Health University of Tours Tours France

**Keywords:** COVID-19 pandemic, digital contact tracing applications, health inequalities, Europe, health promotion

## Abstract

To combat the COVID-19 pandemic, many European countries have developed a public health strategy involving the use of digital contact tracing (DCT) applications to improve timely tracking and contact tracing of COVID-19 cases. France’s independent COVID-19 Control and Society Connection Council (CCL) was established by law in May 2020 to issue advice and recommendations on the national epidemic digital systems. In this paper, we present the recommendations by the CCL, with the objective to increase the uptake and utility of French DCT applications. As the country's most vulnerable population has been subjected to greater virus exposure, a stronger impact of the lockdown, and less access to preventive and health care services, the CCL is particularly aware of health inequalities. The French DCT app TousAntiCovid had been downloaded by 13.6 million users (ie, 20% of the French population) in March 2021. To promote the use of DCT apps, the CCL has recommended that communication about the app’s individual and collective objectives be increased. The CCL has also recommended the introduction of clear, simple, accessible, incentivizing, noncoercive information within the digital tools. In addition, the CCL has recommended improving public health policies to address the needs of the underprivileged. The CCL calls for promoting population empowerment with the use of digital tools, improving public health culture for decision-makers dealing with health determinants, taking social considerations into account, and incorporating community participation.

## Introduction

To combat the first wave of the COVID-19 pandemic, many European countries instituted a strict lockdown coupled with reverse-transcription polymerase chain reaction (RT-PCR) testing, which led to a relative decline in case fatality [1]. These countries also developed a public health strategy involving the use of digital contact tracing (DCT) applications to improve timely tracking and contact tracing [2,3]. Despite these measures, the second epidemic wave occurred in autumn 2020, more rapidly and with a higher severity than expected, leading to a second lockdown in many countries. France developed a national “Test, Trace and Isolate” strategy, but did not achieve its goal of less than 5000 confirmed COVID-19 cases per day by December 15, 2020. As of that date, incidence had increased, and a curfew went into effect first. Several weeks later, in facing a third wave, a lockdown was reinstituted by many countries.

## Recommendations for DCT Use in France

France’s independent COVID-19 Control and Society Connection Council (CCL) was established by law in May 2020 in order to issue advice and recommendations on the national epidemic digital systems. The CCL comprises 13 members, including representatives from the parliament and civil society, patient organizations, jurists, and academics from different disciplines (see Multimedia Appendix 1 [4]). Considering the characteristics of European DCT apps and their potential, we present the CCL’s recommendations, with the objective to increase the uptake and utility of French DCT apps. As the country's most vulnerable population has been subjected to greater virus exposure, a stronger impact of the lockdown, and less access to preventive and health care services, the CCL is particularly aware of prevailing health inequalities.

## Findings

Several mobile apps for contact tracing and information on public health policy have been developed in Europe (Table 1). These apps should respond to scientifically valid and time-bound ethical guidelines. Insufficient privacy protection could erode trust in the government and public health services [5,6]. Unfortunately, adoption of these apps has been below expectations [7]; although, adoption has been high in Finland (45%), it has been limited elsewhere (24% in Ireland, 21% in Switzerland, 19% in Germany, and 13% in Italy [8]; see Table 1). The French DCT *StopCovid* app was based on Bluetooth-based exposure notification. Conceived as a silent app (ie, without interactivity), it was replaced on October 22, 2020, by a conversational app renamed *TousAntiCovid*. Among 66 million inhabitants, only 1.5 million (2.3%) had downloaded the *StopCovid* app and, as of March 2021, this number increased to 13.6 million (ie, 20% of the French population). Moreover, positive test declarations (n=183,377), such as notifications of a contact (n=107,465) have been weak. The poor results of the StopCovid app stemmed from the lack of attractiveness and prevention information, a fear of insufficient privacy protection, Bluetooth connection problems, and the incompatibility of apps with older smartphones.

To promote the use of the apps, the CCL recommended increasing the communication about the individual and collective objectives of the *TousAntiCovid* app. The CCL also recommended the introduction of clear, simple, accessible, incentivizing, noncoercive information in the digital tool. Indeed, the messages aimed at emphasizing risk are less effective than those encouraging self- and collective efficacy [9]. Moreover, to stimulate DCT uptake, apps should enhance perceived benefits [10]. In addition, the CCL recommended improving public health policies to address the needs of the underprivileged population [11]. It bears mentioning that apps are effective only if users are comfortable with digital tools; technical problems are among the main reasons for failure to download [12]. It is also worth noting that the COVID-19 pandemic has accelerated digital health uptake: 49% of the French population used their first health digital tool during the first lockdown [13], whereas 16% of French population never connect to internet [14].

The CCL believes that the national “Test, Trace and Isolate” strategy needed consistency—testing without effective tracing and efficient isolation of positive cases makes little, if any, sense. In Europe and France, apps have progressively incorporated an isolation module (questions about needs during isolation). Their development should be aimed at measuring isolation prescription and adherence. Apps could help families to stay connected and maintain links to resources that support their physical and mental well-being [15]. Despite contrasting strategies of lockdown, no dramatic difference in the magnitude of the second epidemic wave seems to have been observed among these countries (Table 1). For persons with positive test results, the strategy adopted in most countries included a mandatory 7-day isolation until newer variants of SARS-CoV-2 were discovered [16].

**Table 1:** European data on COVID-19 mortality, government stringency responses, and digital contact tracing (DCT) applications as of December 15, 2020.

European country	Population	Unstandardized COVID-19 mortality (per million inhabitants)	Government response stringency index^a^ [17]	DCT app name	Data destruction apps data^b^	Technology	Technology	Uptake rate (%)
Finland	5,540,720	83.2	44.91	Koronavilkku	Yes	Bluetooth	Google/Apple	45
Denmark	5,792,202	164.0	45.37	Smittestopp	Yes	Bluetooth	Google/Apple	—^c^
Switzerland	8,654,622	643.8	46.30	SwissCovid	Yes	Bluetooth	Google/Apple DP3T^d^	21
Estonia	1,326,535	116.1	48.15	Estonia’s App	No	Bluetooth	Google/Apple	—
Norway	5,421,241	72.5	52.78	Smittestopp	Yes	Bluetooth and location	Other	—
United Kingdom	67,886,011	948.7	54.17	NHSCovid19	Yes	Bluetooth	Google/Apple	—
Netherlands	17,134,872	587.9	56.48	Coronamelder	Yes	Bluetooth	Google/Apple	—
Bulgaria	6,948,445	840.2	57.41	ViruSafe	Yes	Location	Other	—
Belgium	11,589,623	1557.8	60.19	Coronoalert	Yes	Bluetooth	Google/Apple DP3T	—
Germany	83,783,942	268.2	67.59	Corona-Warn-App	Yes	Bluetooth	Google/Apple	19
Cyprus	1,207,359	67.9	69.44	CovTracer	Yes	Location	Other	—
Poland	37,846,611	615.9	71.30	ProteGO	Yes	Bluetooth	Google/Apple	—
Spain	46,754,778	1026.9	71.30	RadarCOVID	Yes	Bluetooth	Google/Apple DP3T	—
Ireland	4,937,786	430.6	72.22	Covid Tracker	Yes	Bluetooth	Google/Apple	24
Hungary	9,660,351	749.1	72.22	VirusRadar	Yes	Bluetooth	Other	—
France	65,273,511	887.2	75.00	TousAntiCovid	Yes	Bluetooth	Other	19
Portugal	10,196,709	554.0	77.78	Stay away Covid	Yes	Bluetooth	Google/Apple DP3T	—
Italy	60,461,826	1075.2	79.63	Immuni	No	Bluetooth	Google/Apple	13
Austria	9,006,398	500.8	82.41^e^	Stopp Corona	Yes	Bluetooth	Google/Apple	—

^a^The Government Stringency Index is a composite measure based on 9 response indicators, including school closures, workplace closures, and travel bans, rescaled to a value ranging from 0 to 100 (100=strictest).

^b^Data were extracted several days after their acquisition.

^c^Not available.

^d^DP3T: decentralized privacy-preserving proximity tracing.

^e^Data as of December 8, 2020.

## Discussion

There are several challenges in motivating populations to download DCT apps, even though it is a promising tool. Between European policies of isolation, which are either strict and instituted by national regulations with severe fines or based on incentives, the CCL recommends an intermediate position with national guidelines accompanied by strong incentive supports, ensuring good adherence from the population and avoiding the weakening of the “testing and contact tracing” facets of the national strategy. However, apps will not by themselves resolve the problem of isolation nonadherence, particularly among persons who are outside the health care system. The CCL recommends adaptations of the informational process for these persons. With regard to those for whom pragmatic isolation is difficult, because of home overcrowding, family composition, or work needs, the CCL recommends a prosocial approach with community officers who ensure that sufficient supplies (eg, essentials such as food, medication, and childcare resources) are provided [18].

To conclude, the CCL calls for promoting empowerment of the population with digital tools, improving public health culture for decision-makers dealing with health determinants, taking social considerations into account, and incorporating community participation [19].

## Appendix

Multimedia Appendix 1 Description of the French COVID-19 Control and Society Connection Council.

## Abbreviations

CCL: COVID-19 Control and Society Connection Council
DCT: digital contact tracing
RT-PCR: reverse-transcription polymerase chain reaction
