# Supplementary material for: Positioning Digital Tracing Applications in the Management of the COVID-19 Pandemic in France
Source: J Med Internet Res. 2021 Oct 7;23(10):e27301. doi: 10.2196/27301 (PMC8500347; doi:10.2196/27301)
Supplement: Multimedia Appendix 1 [file jmir_v23i10e27301_app1.docx]

**Multimedia Appendix 1: The independent French COVID-19 Control and Society Connection Council (CCL)**

The CCL has a decision-making support role for the French Minister of Solidarity and Health. It provides consultative, simple and independent advices to the Government.

Through regular audits, the CCL:

1. assess the digital tools contribution in the epidemic = to determine whether digital tools are likely to make, or not, a significant difference in the epidemic management through health team’s feedback
2. verify the medical secrecy respect and the personal data protection.

According to the law (décret no. 2020-572 du 15 mai 2020) [4], the CCL is composed by:

- 4 members of the Parliament
- 1 member of the National Health Conference
- 1 member of the National Council of the Physicians’ college
- 1 member of the French Scientific Council
- 1 member of the National Council of Medical Biology
- 1 member of National Digital Council
- 1 member of the National Consultative Ethics Committee for health and life sciences
- 1 member of the French Public Health Society
- 2 members of the France Assos Santé.

To fill its mission, the CCL establish its own agenda meetings and meet as often as is necessary. It delivers opinions adopted in a collegial manner or may deliver differing opinions of his members.
